# Predicting COVID-19 Patient Disposition Using the National Early Warning Score 2: A Retrospective Cohort Study

**DOI:** 10.7759/cureus.79610

**Published:** 2025-02-25

**Authors:** Asmaa Haj Husin, Hind Ahrari, Jeffrey Keep

**Affiliations:** 1 Medicine, Mohammed Bin Rashid University of Medicine and Health Sciences, Dubai, ARE; 2 Emergency, Mediclinic Parkview Hospital, Dubai, ARE

**Keywords:** care disposition, covid 19, covid prognosis, emergency department, news 2 score

## Abstract

Objectives

To evaluate the effectiveness of the National Early Warning Score 2 (NEWS2) in predicting the need for hospital admission and close monitoring of suspected patients with COVID-19 presenting to the Emergency Department (ED). This study aims to determine whether NEWS2 can aid in identifying high-risk patients with COVID-19 requiring urgent care and admission.

Methodology

Retrospective data from electronic health records of 300 patients with COVID-19 who presented to Mediclinic Parkview Hospital ED between January 1, 2021, and June 30, 2021, were analyzed. Collected variables included age, gender, body mass index (BMI), vital signs, and patient disposition. Statistical analysis was conducted to assess the ability of NEWS2 to predict COVID-19 patient disposition.

Results

A total of 300 patients were included, and their NEWS2 scores were analyzed to predict clinical deterioration. NEWS2, with a cutoff value of 2, predicted hospital admission with 86% sensitivity and 75% specificity. It achieved an average area under the curve (AUC) of 0.86 for predicting outcomes at 24 to 72 hours from the time of initial presentation to the ED.

Conclusions

NEWS2 demonstrates high sensitivity in predicting the disposition of patients with COVID-19. Our findings support the use of NEWS2 as a useful tool for the initial assessment of patients with COVID-19 presenting to the ED, assisting in identifying patients at risk of deterioration.

## Introduction

In December 2019, a novel enveloped RNA beta-coronavirus, later named SARS-CoV-2, was first reported in Wuhan, China [1]. COVID-19 spread across and outside China rapidly and was declared a pandemic on the 11th of March, 2020 challenging healthcare systems. The COVID-19 pandemic spread throughout the world as a severe acute respiratory disease. The pandemic has led to a dramatic loss of human life and has been straining healthcare systems worldwide [2]. As of January 5, 2025, 777 million cases have been reported worldwide, including more than 7 million COVID-19-related deaths [3]. Typical patients with COVID-19 present with fever, myalgia, fatigue, and dry cough. Patients admitted to the hospital can develop severe disease with life-threatening respiratory and multi-organ failure and a high risk of mortality [4].

Since the beginning of the pandemic, hospital visits initially surged, placing significant pressure on medical staff. While many countries have made substantial progress in managing COVID-19, the ongoing unpredictability of patient surges and the potential for new variants highlight the need for healthcare systems to remain prepared for future challenges. This study is important as it helps identify tools that can assist in managing patient flow and decision-making. Healthcare systems must, therefore, ensure that patients with alarming early warning scores obtain the needed hospital care to avoid adverse outcomes. Due to the overwhelming demand for medical examination and triage in Emergency Departments (EDs), the National Early Warning Score (NEWS) could be utilized as a tool to ensure safe decision-making under these stressful circumstances. In times of health crisis and epidemics, clinical scores are often used to rapidly and accurately assess patients. The early warning score system (EWSS) was designed to allocate points for patients’ multiple vital signs in a quantitative manner and assess the sum of each point. In 2012, the Royal College of Physicians recommended standardizing the Early Warning Score System (EWSS) for the National Health Service, which became known as NEWS. NEWS improves the detection of and response to clinical deterioration [5]. It provides a standardized approach to assessing acute illness, incorporating multiple variables such as respiration rate, systolic blood pressure, heart rate, oxygen saturation, supplemental oxygen use, temperature, and level of consciousness (Alert [A], Voice [V], Pain [P], Unresponsive [U]). These variables are routinely collected by nursing staff when patients present to the ED [6]. Although previous research in Norway suggested using NEWS as a prognostic factor, no studies have been conducted in UAE [7]. Moreover, little is known about differences in admissions patterns among patient groups with COVID-19. The Swiss Society of Intensive Care Medicine has recommended the use of EWS parameters for the admission of patients; however, this recommendation is based on previous evidence of patients without COVID-19 [8].

This observational, retrospective study aims to design a guideline that can reduce the existing pressure on physicians by providing a standardized, evidence-based tool (NEWS2) to quickly and accurately triage patients with COVID-19. This can assist physicians in prioritizing care, reducing decision-making time, and ensuring timely interventions, ultimately improving workflow in high-pressure situations. Vitals and sociodemographic variables of patients with COVID-19 were collected from medical records in the ED of Mediclinic Parkview Hospital, and their NEWS2 was subsequently calculated. The NEWS2 parameters were then analyzed to allow for the categorization of patients based on their health status and their expected disposition and clinical deterioration. We predict that the NEWS2 score may be a useful marker in predicting the prognosis and disposition of patients with COVID-19.

## Materials and methods

Study design

This study was a retrospective cohort study. This retrospective cohort study analyzed confirmed patients with COVID-19 who presented to the ED between January 1, 2021, and June 30, 2021. The study was analyzed and discussed using Strengthening the Reporting of Observational Studies in Epidemiology (STROBE) guidelines (Appendix A) [9].

Ethical considerations

This study was approved by the Institutional Review Board of Mohammed Bin Rashid University of Medicine and Health Sciences (MBRU-IRB). All patient data were anonymized and managed per ethical guidelines.

Study setting

Data were extracted from a tertiary care hospital in Dubai, UAE. Mediclinic Parkview Hospital, an 182-bed general hospital, included a large ambulatory care component with over 120 exam and treatment rooms. The hospital electronically recorded patients’ vitals at presentation as part of the standard care process, enabling the calculation of NEWS2.

Study participants

The hospital confirmed COVID-19 infection through the qualitative detection of SARS-CoV-2 nucleic acid using polymerase chain reaction (PCR) testing on nasal swabs [10]. Both male and female adult patients (>18 years of age) with a positive COVID-19 result and electronic NEWS2 parameters recorded within ±24 hours of admission were considered participants in this study. Patients with confirmed COVID-19 infection who presented to the ED for other reasons, such as surgical procedures, were also included. Minors (<18 years of age) and pregnant women were excluded. Additionally, patients with a positive COVID-19 result who were referred to another hospital within the first 24 hours of presentation were excluded. Patients with missing vital sign parameters required for NEWS2 calculation were excluded from the study to ensure data accuracy and reliability. No imputation methods were applied.

Study variables

For each patient with a positive COVID-19 result, we obtained a pseudonymized patient identifier, age (in years), gender (male/female), body mass index (BMI), vital signs (including respiratory rate, temperature, systolic blood pressure, heart rate, oxygen saturation, oxygen supplementation, and level of consciousness [AVPU]), and the patient's final disposition. Vital signs were measured by ED nurses trained to adhere to standard operating procedures (SOPs) for vital sign measurement. Despite these measures, variability existed in these parameters, especially in the AVPU scoring, which included subjective components. The outcome variable was the NEWS2, calculated using the recorded vital signs.

Data sources/measurement

**Figure 1:**
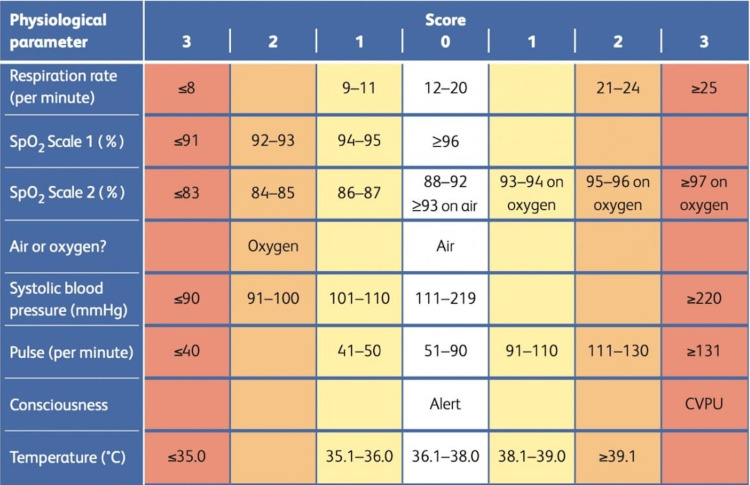
The NEWS2 scoring system. Source: [5]. NEWS2, National Early Warning Score 2

The primary source of data collection for all variables was the electronic health records (EHRs) of Mediclinic Parkview Hospital. All EHRs were accessed by password-protected devices. Patient and vitals evaluations were obtained by the ED triage nurse. NEWS2 parameters, as shown in Figure 1, were body temperature (in °C), respiratory rate (per minute), heart rate (per minute), systolic blood pressure (mmHg), supplemental oxygen, saturation of percutaneous oxygen (SpO2) (%), and level of consciousness (AVPU). Parameters were rearranged based on NEWS2 groupings. They were scored from 0 to 3 points each, except oxygen saturation which ranges from 0 to 2 points [11]. A higher score indicates that the parameter is further from the normal range. Admitted patients’ length of stay and level of care (ICU, Ward, ED) were recorded and taken into consideration

Statistical methods

The research data were entered into Excel and analyzed using the SPSS software (version 24; IBM Corp., Armonk, NY) to ensure data quality. A range check was performed using histograms and frequency distributions for the study variables. Our sample of 300 patients with COVID-19 included patients with different dispositions. Sensitivity/specificity analysis and receiver operating characteristic (ROC) curves were used to investigate NEWS2 performance and detect the cutoff value for NEWS2 as a predictor of patients’ disposition. The ROC curve plots sensitivity (true positive rate) against 1-specificity (false positive rate) for consecutive predicted risks [12]. The area under the ROC curve (AUC) was used to measure the discriminative ability of NEWS2. Mean, median, and mode were calculated for sociodemographic variables, including age and BMI. A gender frequency table was created, and differences in age and BMI between the two gender categories were analyzed. The *P*-value of the sociodemographic variables was analyzed using the chi-square test and t-test to determine statistical significance. A *P*-value of ≤0.05 was considered statistically significant.

## Results

Participants’ characteristics

A total of 300 patients with COVID-19 presented to the ED with recorded vital signs during the analysis period. The mean ± standard deviation (SD) age of patients was 39.5 ± 10.8 years, with 6.3% aged above 60 years. The mean ± SD of BMI was 27.1 ± 5.1. Most patients were male (153/300, 51%). The mean ± SD age was 41 ± 10 years for males and 38 ± 11 years for females. The mean ± SD BMI was 28.0 ± 5.2 for males and 26.1 ± 4.8 for females. The study participants represented more than 38% of the total ED population during the study period. A total of 22 patients (7.3%) were admitted to the hospital, while the remaining patients were sent for home isolation for further recovery. In our sample, 13 patients (4.3%) were admitted to the COVID-19 ward, while 9 patients (3%) were admitted to the ICU. Patients admitted to the COVID-19 ward had a mean ± SD age of 57 ± 14.4 years and were predominantly female (8/13, 61.5%). Patients transferred to the ICU had a mean ± SD age of 61 ± 11.2 years and were predominantly male (8/9, 88.9%).

Outcome data

**Figure 2:**
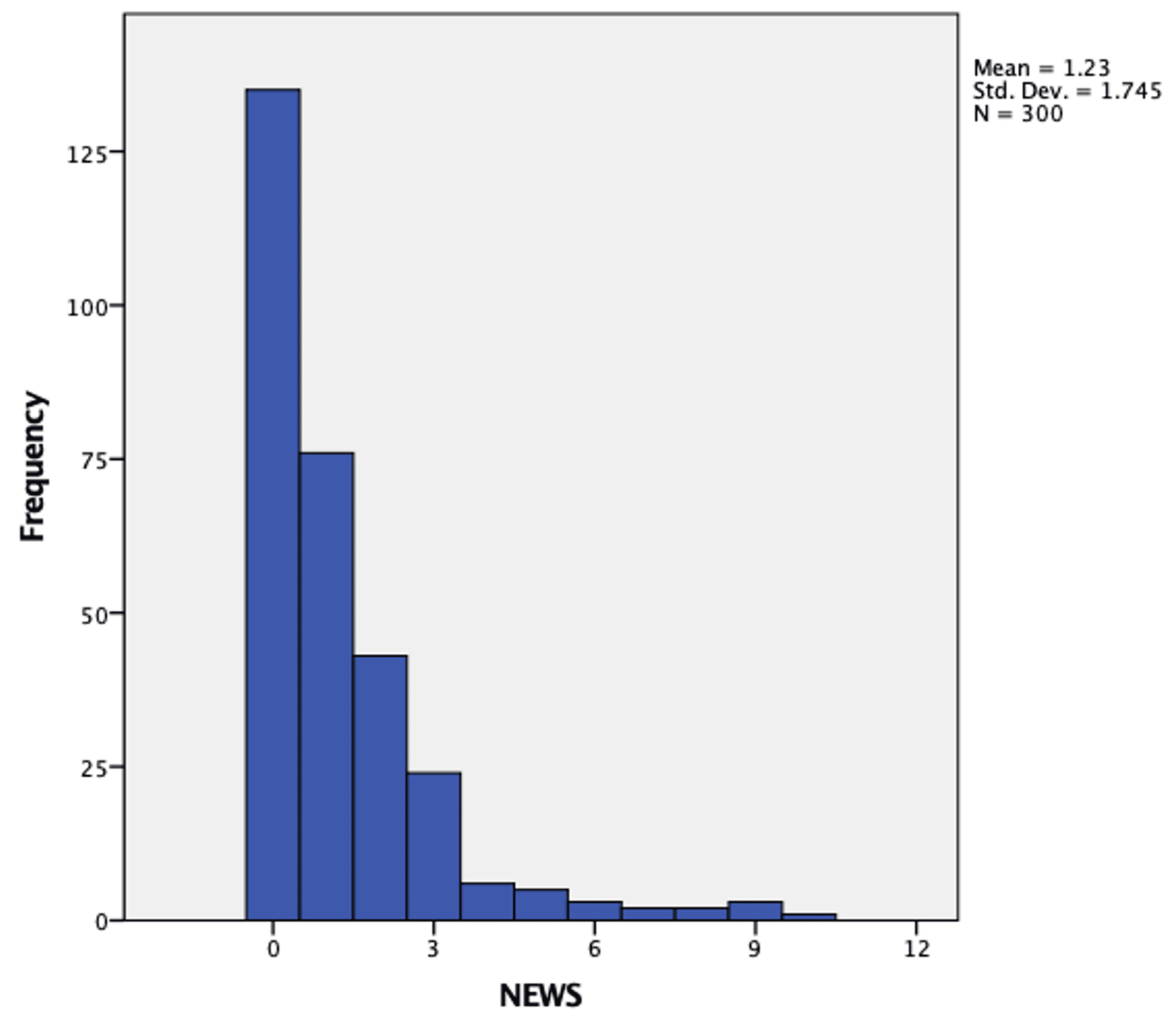
Histogram presenting NEWS2 distribution in the study population. NEWS2, National Early Warning Score 2

Patient details, including NEWS2 and disposition, were collected. The distribution of NEWS2 among the 300 patients is presented in the histogram shown in Figure 2. The NEWS2 score of patients who required admission at the time of presentation was significantly higher than that of patients sent for home isolation, with a median score of 5.5 (interquartile range [IQR], 2.75-8) compared to 1 (IQR, 0-1.25), respectively. This difference was statistically significant (*P*-value ≤ 0.05). The NEWS2 of the patients requiring ICU admission within 24 hours of disposition was higher than that of patients admitted to the COVID-19 ward with a median score of 8 [IQR, 5-9] and median score of 4 [IQR, 2-6], respectively; the difference was statistically significant (*P*-value ≤ 0.05). As shown in Table 1, non-admitted patients were more likely to have a normal temperature between 36.1 and 38.0 °C, whereas admitted patients were more likely to have either a normal or elevated temperature between 38.1 and 39.0 °C. Admitted patients were also more likely to have a heart rate between 91 and 110 bpm, while non-admitted patients were more likely to have a heart rate between 51 and 90 bpm. Both admitted and non-admitted patients were likely to have a systolic blood pressure between 111 and 119 mmHg, a respiratory rate of 12 to 20 breaths per minute, and an oxygen saturation of greater than 96%. Age and sex were also significantly different between the two groups; however, these variables are often considered confounding factors. Nevertheless, age remains a risk factor for severity and death in the context of COVID-19, even after accounting for associated comorbidities [13].

Twenty-two patients from the study sample were admitted, with 13 (59.1%) admitted to the COVID-19 ward and 9 (40.9%) to the ICU. Their length of stay and level of care were recorded. Two patients were admitted to the COVID-19 ward, with a NEWS2 value of 0. One of the patients was an 82-year-old female admitted to the COVID-19 ward for one day due to the risk of deterioration associated with her age. The other patient was a 72-year-old male admitted to the COVID-19 ward for nine days where he was then discharged. He required medical attention due to his age and high BMI (25.95). The patient admitted to the COVID-19 ward with the highest NEWS2 score upon presentation was a 49-year-old male who was transferred to the ICU after two days. He presented to the ED with a NEWS2 value of 8 predominantly due to low oxygen saturation (<91%). The patient admitted to the ICU with the lowest NEWS2 value was a 49-year-old male who stayed in the ICU for 24 days where he then died of severe COVID-19 infection. He presented to the ED with a NEWS2 value of 3 predominantly due to low oxygen saturation (92%). A patient with the highest NEWS2 value seen in the study was sent to the ICU. He was a 79-year-old male who presented to the ED, with a NEWS2 value of 10 being confused, with a high respiratory rate, and low oxygen saturation (<91%). The patient stayed in the ICU for 16 days where he then died of severe COVID-19 infection. 

**Table 1:** Frequency of NEWS2 parameters across admitted and non-admitted patients. NEWS2, National Early Warning Score 2; V, Verbal response; P, Pain response; U, Unresponsive

		Outcome				
		Non-admitted		Admitted		
		Count, *n*	%	Count, *n*	%	
Temperature (°C)	36.1-38	223	96.1%	9	3.9%	
	35.1-36‎ or 38.1-39	53	85.5%	9	14.5%	
	>39.1	2	33.3%	4	66.7%	
	<35	0	0.0%	0	0.0%	
Heart rate (bpm)	51-90	178	96.7%	6	3.3%	
	41-50‎ or 91-110	79	89.8%	9	10.2%	
	111-130‎	21	75.0%	7	25.0%	
	<40‎ or >131	0	0.0%	0	0.0%	
Systolic BP (mmHg)	111-219	230	91.6%	21	8.4%	
	101-110	34	97.1%	1	2.9%	
	91-100	14	100.0%	0	0.0%	
	<90 or >220	0	0.0%	0	0.0%	
Respiratory rate (breaths per minute)	12-20	274	95.1%	14	4.9%	
	9-11	0	0.0%	0	0.0%	
	21-24	4	57.1%	3	42.9%	
	<8‎ or >25	0	0.0%	5	100.0%	
SpO2 (%)	>96	274	97.5%	7	2.5%	
	94-95	3	60.0%	2	40.0%	
	92-93	0	0.0%	1	100.0%	
	<91	1	7.7%	12	92.3%	
Oxygen	Air	277	93.3%	20	6.8%	
	On oxygen	1	33.3%	2	66.7%	
Level of consciousness	Alert	278	93.3%	20	6.7%	
	V, P, U	0	0.0%	2	100.0%	

Main results

**Figure 3:**
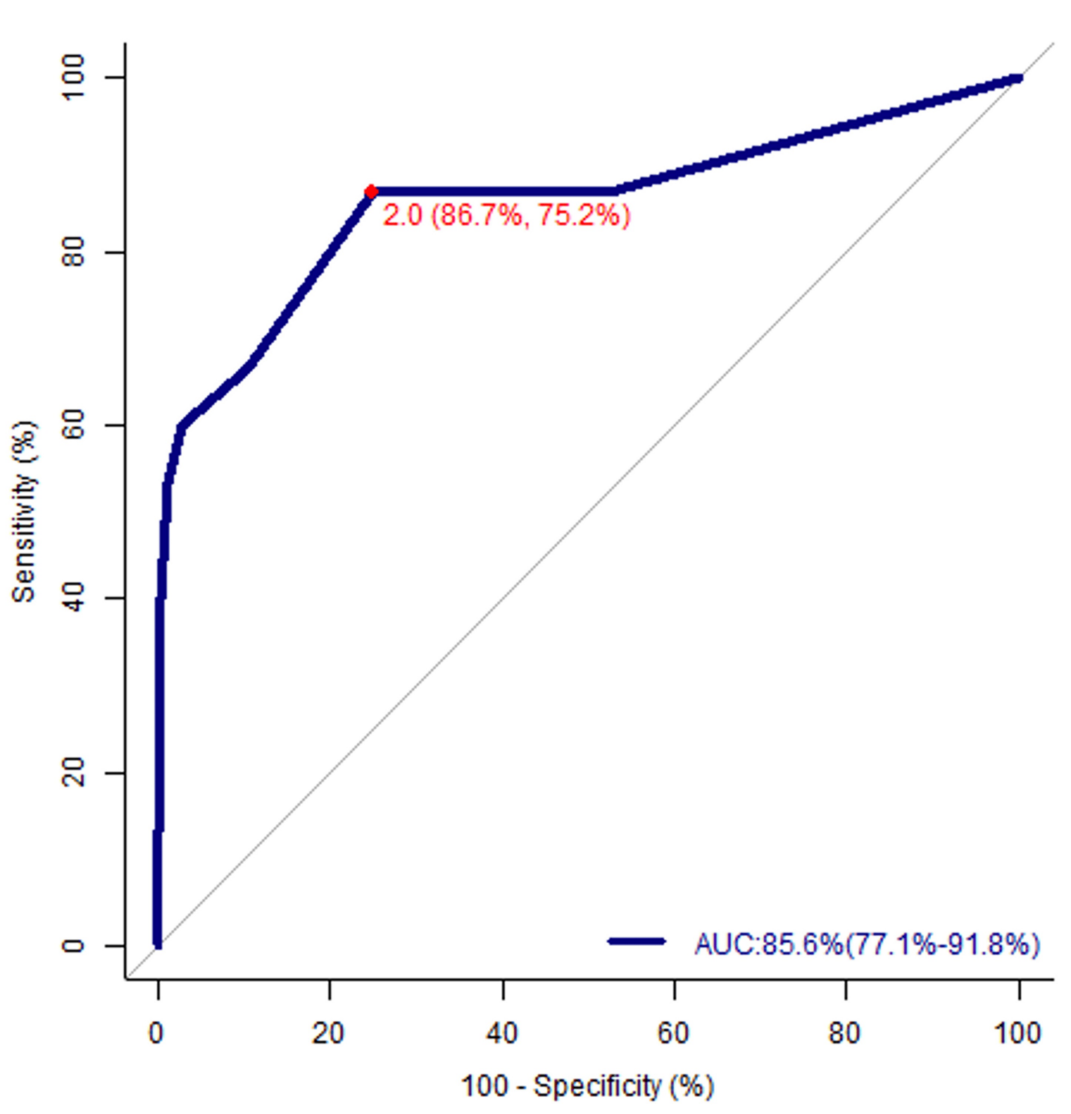
NEWS2 ROC curve. AUC, area under the curve; ROC, receiver operating characteristic; NEWS2, National Early Warning Score 2

Table 2 displays the sensitivity and specificity for all NEWS2 values in patients with COVID-19. A cutoff value of 2 provided the best sensitivity (86%) and specificity (75%). The AUC for NEWS2 was 85.6% (77.1%-91.8%), as shown by the ROC curve in Figure 3, which measured its discriminative ability. With a score of 85.6%, NEWS2 calculated upon arrival to the ED was capable of predicting the disposition and prognosis of patients with COVID-19. The Youden index (J) was 0.618, as shown in Table 3. The percentage of men and women admitted to either the ward or ICU was 51% and 49%, respectively; the 1% difference was statistically significant (*P*-value ≤ 0.05). The mean ± SD age for admission was 41 ± 10 years for males and 38 ± 11 years for females; the three-year difference was statistically significant (*P*-value ≤ 0.05).

**Table 2:** Sensitivity and specificity for all NEWS2 values. NEWS2, National Early Warning Score 2; LR, likelihood ratio; LCL, lower confidence limit; UCL, upper confidence limit

Threshold	Sensitivity (%)	Specificity (%)	LR	LR LCL	LR UCL
≥0	100	0	0.279	0.076	1.019
≥1	86.667	47.842	0	0	1.873
≥2	86.667	75.18	1.39	0.485	3.98
≥3	66.667	89.568	0.883	0.127	6.127
≥4	60	97.122	3.707	0.462	29.766
≥5	53.333	98.921	18.533	2.8	122.661
≥6	40	99.64		5.829	
≥7	20	99.64	18.533	1.218	282.098
≥8	13.333	100		1.294	
≥9	6.667	100		1.294	

**Table 3:** Summary of statistical findings.

Summary	
Area under the ROC curve (AUC)	0.856 (0.771, 0.918)
Standard error	0.067
Z-statistic	5.281
Prob > Z (*P*-value)	<0.001
Youden index (J)	0.618
Cutoff	2
Sensitivity (%)	86.667
Specificity (%)	75.18

## Discussion

The study was based on the collection of data from 300 patients presenting to the ED between January 1, 2021, and June 30, 2021, and the calculation of their NEWS2 within the first 24 hours of presentation. At presentation, the NEWS2 score of patients requiring admission was significantly higher than that of patients in home isolation. An optimal cutoff value of 2 was determined to assist physicians in identifying patients with COVID-19 requiring hospital admission as it achieved the best sensitivity (86%) and specificity (75%). It improves the identification of the appropriate level of care for each patient based on the NEWS2 score calculated at triage in the ED. This will allow for close monitoring of patients at a higher risk of developing severe symptoms and COVID-19-related complications. Since NEWS2 achieved an AUC of 85.6%, the key finding of this study indicates that its use in the ED is effective in predicting the likelihood of hospital admission for patients with COVID-19. The statistical values strongly supported the use of NEWS2 as a sensitive method to initially assess patients with COVID-19 and anticipate their admission to a specialized emergency care unit. 

This study has provided sufficient evidence to support the use of NEWS2 as a potential disposition and prognosis marker; however, this study has several limitations. This research is retrospective, and the ED triage nurses did not collect the vital signs for this study. As a result, some values were missing, further excluding patients from the study. Additionally, variability exists in vital sign measurements among the ED triage nurses, which could influence the accuracy of the measurements. The NEWS2 scoring system relies on objective measures, including temperature, heart rate, blood pressure, and oxygen saturation, and subjective measures, including the respiratory rate and level of consciousness (AVPU). As previously mentioned, nurses were trained to adhere to SOPs; however, the degree of variability among nurses was not formally assessed, representing a limitation. As this study was conducted in a hospital with a low COVID-19 patient load, our findings may not be generalizable to all hospitals nationwide. Different cutoff values might have been obtained if the study had been conducted in a COVID-19-dedicated hospital with different admission criteria and triage systems. The database did not include information on race/ethnicity, socioeconomic status, occupation, and smoking status of patients, which may impact the prognosis of our selected participants. Other limitations include the presence of confounders that cannot be eliminated in an observational study, differences in circulating COVID-19 variants between 2021 and the present, and the lack of consideration for patients' vaccination status.

The high number of males admitted to ICU is related to what had been reported of increased vulnerability of men to COVID-19 concluded by a previous study [14]. A study analyzing 95,180 COVID-19 hospitalizations among patients 18 years and older revealed that in-hospital mortality, prolonged length of hospital stays, vasopressor use, mechanical ventilation, and ICU admission rates were significantly higher among male compared with female hospitalizations [15]. Another study conducted in Italy with 2,378 patients reported that male gender was a primary determinant for ICU admission, with men constituting 74% of ICU admissions [16]. The average age of the patients with COVID-19 in this study with a mean age of 39.5 ± 10.8 years was within the same range of other research conducted in the Gulf region reported in Oman [17] and Kuwait [18]. A recent study involving 403 patients with COVID-19 was conducted, in which NEWS2 and the quick Sepsis-related Organ Failure Assessment (qSOFA) scores were calculated at admission, 24 hours post-admission, and 48 hours post-admission to compare their effectiveness in predicting prognosis. The mean NEWS2 score at admission, 24 hours, and 48 hours post-admission was superior to the qSOFA scores in determining the need for intensive care support and mortality. Higher NEWS2 scores were more valuable and accurate in predicting patients with poor prognoses [19]. These findings reinforce the value of NEWS2 as a reliable early warning score for triaging patients and anticipating their clinical trajectory, particularly in a dynamic setting like an ED. A previous study compared other common scoring systems to NEWS2 and concluded that NEWS2 is the best in predicting the prognosis of respiratory function in patients with COVID-19 [20]. NEWS2 has also proved to be effective in several studies not related to COVID-19 [21,22]. However, the NEWS2 value suggested by this study differs from that reported in other research. The admission rate for patients with a NEWS2 cutoff of ≥2 is relatively low compared to similar research in other regions, which identified a cutoff of ≥5 [14-22]. This reflects the significant level of care provided by the UAE healthcare system, ensuring that even individuals with mild illness receive adequate care to prevent deterioration.

Despite our previously mentioned limitation, the findings are still highly relevant. NEWS2, to this date, is still widely used for triaging patients with respiratory illnesses, including COVID-19 and its complications. The time frame of this study corresponds to a pivotal period early in the pandemic when healthcare systems worldwide were facing unprecedented strain. This makes the study particularly relevant as healthcare providers continue to apply lessons learned from the pandemic and assess the long-term implications of COVID-19 on patient management. Several research [23] targeting patients with COVID-19 have concluded that the mean age of hospitalized patients with COVID-19 is >50 years, and since patients admitted in our study sample have a mean age of 57 years, the study can be taken into consideration by some hospitals with similar admission criteria. Hospitals with settings similar to that of Mediclinic Parkview Hospital can depend on NEWS2 to triage COVID-19 hospitals. In cases with a NEWS2 score is <2, despite the low score, monitoring at regular intervals and repeat calculations of NEWS2 scores can detect a slight rise from the initial score, allowing early detection of possible deterioration in a patient's condition. It can also stratify patients based on their risk of developing complications and allows healthcare workers to observe those patients requiring further care closely.

## Conclusions

This is the first retrospective study suggesting the use of NEWS2 to triage patients with COVID-19 in the UAE. NEWS2 has shown a good predictive capacity and sensitivity in assessing patients with COVID-19. The study has shown that patients presenting to the ED with a NEWS2 value lower than that suggested by other research will require admission. Patients with COVID-19 and a NEWS2 score as low as 2 should be hospitalized within the first 24 hours of their ED presentation to prevent deterioration or rehospitalization if discharged. These findings can help alleviate the stress and pressure placed on health workers during these tough times and facilitate a fast, non-invasive triaging system.

## Appendices

Appendix A 

**Table 4:** STROBE checklist.

Section/Topic	Item #	Recommendation	Reported on page #
Title and abstract	1	(a) Indicate the study’s design with a commonly used term in the title or the abstract	Title, Abstract, paragraph 2
		(b) Provide in the abstract an informative and balanced summary of what was done and what was found	Abstract, paragraph 2-4
Introduction			
Background/rationale	2	Explain the scientific background and rationale for the investigation being reported	Introduction, paragraph 1-3
Objectives	3	State specific objectives, including any pre-specified hypotheses	Introduction, paragraph 3
Methods			
Study design	4	Present key elements of study design early in the paper	Methods, paragraph 1
Setting	5	Describe the setting, locations, and relevant dates, including periods of recruitment, exposure, follow-up, and data collection	Methods, paragraph 2
Participants	6	(a) Cohort study—Give the eligibility criteria, and the sources and methods of selection of participants. Describe methods of follow-up Case-control study—Give the eligibility criteria, and the sources and methods of case ascertainment and control selection. Give the rationale for the choice of cases and controls Cross-sectional study—Give the eligibility criteria, and the sources and methods of selection of participants	Methods, paragraph 3
		(b) Cohort study—For matched studies, give matching criteria and number of exposed and unexposed Case-control study—For matched studies, give matching criteria and the number of controls per case	--
Variables	7	Clearly define all outcomes, exposures, predictors, potential confounders, and effect modifiers. Give diagnostic criteria, if applicable	Methods, paragraph 4
Data sources/measurement	8*	For each variable of interest, give sources of data and details of methods of assessment (measurement). Describe comparability of assessment methods if there is more than one group	Methods, paragraph 5
Bias	9	Describe any efforts to address potential sources of bias	--
Study size	10	Explain how the study size was arrived at	Methods, paragraph 6
Quantitative variables	11	Explain how quantitative variables were handled in the analyses. If applicable, describe which groupings were chosen and why	Methods, paragraph 6
Statistical methods	12	(a) Describe all statistical methods, including those used to control for confounding	Methods, paragraph 6
		(b) Describe any methods used to examine subgroups and interactions	Methods, paragraph 6
		(c) Explain how missing data were addressed	--
		(d) Cohort study—If applicable, explain how loss to follow-up was addressed Case-control study—If applicable, explain how matching of cases and controls was addressed Cross-sectional study—If applicable, describe analytical methods taking account of sampling strategy	Methods, paragraph 6
		(e) Describe any sensitivity analyses	Methods, paragraph 6
Results			
Participants	13*	(a) Report numbers of individuals at each stage of study—eg numbers potentially eligible, examined for eligibility, confirmed eligible, included in the study, completing follow-up, and analysed	Results, paragraph 1
		(b) Give reasons for non-participation at each stage	Results, paragraph 1
		(c) Consider use of a flow diagram	--
Descriptive data	14*	(a) Give characteristics of study participants (eg demographic, clinical, social) and information on exposures and potential confounders	Results, paragraph 1
		(b) Indicate number of participants with missing data for each variable of interest	Results, paragraph 1-2
		(c) Cohort study—Summarise follow-up time (eg, average and total amount)	--
Outcome data	15*	Cohort study—Report numbers of outcome events or summary measures over time	--
		Case-control study—Report numbers in each exposure category, or summary measures of exposure	--
		Cross-sectional study—Report numbers of outcome events or summary measures	--
Main results	16	(a) Give unadjusted estimates and, if applicable, confounder-adjusted estimates and their precision (eg, 95% confidence interval). Make clear which confounders were adjusted for and why they were included	Results, paragraph 3
		(b) Report category boundaries when continuous variables were categorized	--
		(c) If relevant, consider translating estimates of relative risk into absolute risk for a meaningful time period	--
Other analyses	17	Report other analyses done—eg analyses of subgroups and interactions, and sensitivity analyses	Results, paragraph 3
Discussion			
Key results	18	Summarise key results with reference to study objectives	Discussion, paragraph 1
Limitations	19	Discuss limitations of the study, taking into account sources of potential bias or imprecision. Discuss both direction and magnitude of any potential bias	Discussion, paragraph 2
Interpretation	20	Give a cautious overall interpretation of results considering objectives, limitations, multiplicity of analyses, results from similar studies, and other relevant evidence	Discussion, paragraph 3
Generalizability	21	Discuss the generalizability (external validity) of the study results	Discussion, paragraph 4
Other information			
Funding	22	Give the source of funding and the role of the funders for the present study and, if applicable, for the original study on which the present article is based	Funding Section
